# Assessing the quality of studies funded by the Israel National Institute for Health Policy Research, 2010–2020

**DOI:** 10.1186/s13584-025-00672-w

**Published:** 2025-03-05

**Authors:** Dan Even, Moshe Leshno, Avi Porath

**Affiliations:** 1 Moshe Prywes Center for Medical Education, Faculty of Health Sciences, Ben-Gurion University of the Negev, P.O. Box 653, 8410501 Beer Sheva, Israel; 2 Coller School of Management, Tel Aviv University, Tel Aviv, Israel; 3 Faculty of Health Sciences, Ben Gurion University of the Negev, Beer Sheva, Israel

**Keywords:** Health policy research, Quality assessment, Societal impact, Publication metrics

## Abstract

**Background:**

Research is the basis of advancement in health and wellbeing in modern societies. Our study aims to examine the funding policy of the Israel National Institute for Health Policy Research (NIHP), a national foundation responsible for assessing the impact of the national Health Insurance Law on health services in Israel. The study aims to evaluate the studies funded from 2010 to 2020, considering their publication in scientific literature and other channels that may influence decision-makers. We compare findings to a previous internal examination of studies funded by the NIHP during 1996–2014. Our paper presents an approach for measuring the impact of health policy research.

**Methods:**

All 378 studies funded by NIHP during the specified years were identified. Objective data were gathered by investigating scientific literature across three datasets: Web of Science (WOS), PubMed, and Google Scholar, including journal impact factor, quarterly index, and citation metrics. Concurrently, a questionnaire was developed to collect additional and subjective data from principal investigators of the funded research projects.

**Results:**

In the final sample of 364 studies funded by NIHP from 2010 to 2020, after 11 were cancelled, and 3 were duplicates. 436 publications were retrieved in peer-reviewed journals. The average time elapsed from funding to scientific publication was 4.65 years. Metric parameters for the top publications of 231 funded studies with at least one publication in peer-reviewed journals revealed an average journal impact factor of 5.97 points and an average of 7.82 citations according to WOS and 14 citations according to Google Scholar. A comparison to 459 funded studies from 1996 to 2014 found a twofold increase in the impact factor. Nearly half of the principal investigators reported some influence on policy processes in the questionnaires, and the majority of the studies were also reported in popular media outlets.

**Conclusions:**

The study provides an overview of the quality and potential influence of studies funded by NIHP, dedicated to supporting research in the field of health policy in Israel. Some of the findings are supported by results from similar inquiries. Several recommendations are introduced to enhance the quality and impact of the funded studies.

**Supplementary Information:**

The online version contains supplementary material available at 10.1186/s13584-025-00672-w.

## Background

Critical review of core activities is a vital component of organizations dedicated to improving public health [1]. Importantly, national organizations that support studies in the field of health policy are required to examine the impact of their funding policies. However, only a limited number of empirical studies have delved into the mechanisms through which funded studies on health policy topics influence policy and practice [2].

The questions of how to measure, evaluate, and maximize investments in health and medical research are significant and relevant not only for the types of knowledge assessed but also for aspects related to health policy. Such analysis can aid in accounting for funds, advocating for additional resources, and discovering better ways to allocate resources more effectively to support studies likely to achieve national strategic objectives [3]. Although the necessity to measure effects of grants has been widely acknowledged by funding institutions, the entire field is still in the theoretical and pragmatic stages of development [4].

In many countries, governments and private research bodies aim to maximize the benefits of their studies, encompassing their extensive effects on the population in medical, health, economic, social, and other aspects. This focus has resulted in the development of Research Impact Assessment Frameworks (RIAFs), providing a structured approach for conceptually evaluating the effects of funded research [5–7]. Some researchers have also proposed checklist platforms for assessing the quality of studies, such as the CHEERS checklist for papers reporting health economic evaluations [8].

At the core of evaluating the impact of researches in the fields of medicine and health lies the scientific publication of the research. Several measures have been proposed to assess the evaluation of studies based on publications, thus focusing on publication metrics, including the number of publications in peer-reviewed journals; impact factor of journals; number of citations of publications and the journal citation index; requests for reprints; contribution of methodological research methods; inclusion in reviews and meta-analyses; and research-led deliverables and patents [9]. Another significant aspect pertains to a broader impact of researches on policy processes, examining their economics, societal, and cultural contributions [4, 10–13].

Beyond economic influence, Kuruvilla et al. highlight the formulation of four central measures that can be used to assess the effects of medical and health studies: research-related, policy, service (health and intersectoral), and societal impacts [14]. Other models have been developed by the International School on Research Impact Assessment (ISRIA) [15], the Canadian Academy of Health Sciences (CAHS) [16], and the U.K. higher education institutes [17].

In recent years, a new field has emerged as particularly relevant to analyzing the impact of health policy studies—the socio-economic assessment of studies. Based on this field, in 2019, a joint project of the European Union and the Hungarian government introduced six channels for assessing the impacts of research infrastructures. The model includes not only the scientific, technological, and economic impacts, but also the social impact (contribution to family and community well-being), political impact, and environmental impact. To collect data, the model suggests utilizing bibliometrics, statistical reports, surveys, evidence reviews, interviews, and analysis of media publications, among other methods [18].

Several organizations funding research in the fields of medicine and healthcare have recently started setting target goals in advance for the future evaluation of the quality of studies. As such an example, funders have recently started publishing RIAFs in their portfolios [6]. In the Australian government, the agenda for supporting scientific research ensures that, for the first time, clear and transparent measures of non-academic influences and industry involvement are introduced while assessing the quality of academic research [19].

Measuring the effects of medical and health funded researches faces several barriers. For example, there are concerns that such measuring may hinder the promotion of basic science and the essential role of scientific knowledge in its early stages. Additionally, measuring incentives may create conflicts with research priorities, such as career advancement and student learning, which are central to university goals. Administrative burden and costs for collecting information and conducting assessments are also challenging. Moreover, the field of assessing the effects of studies is yet to be fully matured [10, 20].

Performance monitoring of health organizations, including the impact of funded research, has garnered increased attention in recent years, especially after the 2008 financial crisis, however there is still no universally recognized standardized methodology for development and implementation of quality assessment tools [5, 6, 21]. Moreover, there is a lack of agreed-upon systematic approaches for measuring such impacts [22].

New models developed to evaluate the activities of national health research institutes require the construction of a new and broader database, which includes information on funded researches from a systemic and social perspective.

Our study introduces a platform for such an analysis, aiming to assess the quality of funded research by the degree of publication in peer-reviewed academic journals within funded research in the Israeli National Institute for Health Policy Research (NIHP).

The NIHP is an Israeli national foundation responsible for assessing the impact of the national Health Insurance Law on health services in Israel. Its primary goal is to accompany the implementation of the National Health Insurance Law and assess its impact on health services in Israel, focusing on their quality, efficiency, and costs. Under the law, national health insurance in Israel shall be based on the principles of justice, equality and mutual assistance [23]. The NIHP promotes interdisciplinary and multi-professional scientific cooperation at a national level among all entities, both managerial and academic, with interests in promoting the Israeli health system. The Institute’s budget is used to fund research according to the Institute's mission. The Institute has no private funding sources in addition to the government source [24].

As part of its activities, the Institute has set itself the goal of periodically evaluating the quality of its decisions in selecting and funding studies, considering their publication in scientific literature and other channels which may influence decision-makers.

## Methods

The study conducted a broad analysis of data concerning the quality of studies funded by the NIHP from 2010 to 2020. This involved identifying objective information in the scientific and professional literature and administering research questionnaires to the researchers.

### Funding policy

The researches supported by the Institute address three central domains in the Israeli healthcare system: the organization of health services, health economics, and the quality of health services, focusing on issues that may contribute in decision-making and policy-making processes. In addition to these general outlines, almost each years the NIHP publishes a “call for proposals”, prioritizing the support of research proposals on specific topics. The relevant proposals undergo peer review in Israel and abroad, with the final decision to support research funding made by the institute’s Research Committee [24]. The requested research budget significantly influences the decision-making process. A standard study typically spans up to a year and a half with a budget ranging from 50,000 to 150,000 Israeli currency of New Shekels (NIS). A more extensive study may last two to three years with a budget not exceeding 300,000 NIS. In the Israeli NIHP, funding research does not require the publication of results in a scientific journal. Our research, conducted in two phases since the establishment of NIHP, focuses on examining the outcomes of studies funded by the organization. This study evaluates research funded through calls for proposals from 2010 to 2020, with some findings compared to a previous internal assessment of studies funded from 1996 to 2014, conducted by one of the current investigators (ML). One of the main evaluation tools used is the assessment of the quality of the scientific journals in which the Institute’s funded research is published. Additionally, we examine other publications and investigate the impact of the studies on decision-makers, based on reports from the researchers themselves.

### Study design

The study commenced with an initial meeting at the National Institute’s offices on May 5, 2022. A database containing all 378 studies funded by the NIHP from January 2010 to December 2020 and completed by May 2022 was prepared, including relevant data such as the study title; names of researchers, including the responsible principal investigator and additional researchers; academic degrees of researchers and their organizational affiliations; number of studies and the year of approval; the institution where the research was conducted; duration of the study; research status; and the amount of funding provided by the NIHP. There were 69 principal investigators with more than one study, therefore, we selected a method that analyzed the data by treating each funded study project as a unit of analysis. The dataset did not include data on the gender of the principal investigators.

Objective data was collected while investigating scientific literature across three datasets: Web of Science (WOS), PubMed, and Google Scholar. WOS, operated by Clarivate, presents citations from over 21,000 peer-reviewed journals worldwide across 254 research fields [25]. PubMed, a free resource developed and maintained by the U.S. NIH, facilitates the search and retrieval of biomedical and life sciences literature, including the Medline database with citations from over 5200 scientific journals in about 40 languages [26]. Google Scholar, a research engine based on the Google platform, provides access to a broad range of scientific journals and citations. In these databases, a proactive search was conducted for published studies related to the research title and/or summary, based on the names of the researchers associated with the funded research proposals. This analysis was conducted from August 2022 to December 2022.

Concurrently, a questionnaire was developed to collect additional data and subjective assessment from principal investigators of the funded research projects. The questionnaire, comprising 32 questions, was formulated with assistance from two health policy professionals, based on a prior internal examination covering 459 studies funded by the NIHP from 1996 to 2014. The questions aimed to detail all types of publications resulting from the studies, including those in peer-reviewed journals, books, book chapters, scientific conferences, independent publications, opinions, research meetings and seminars, and publications in popular and social media platforms. Additionally, the questionnaire assessed each principal investigator's perception of the research's impact on decision-making processes within the Israeli health system and its broader effects on medical, economic, and social domains. The questionnaire was prepared using the FORMS application of Microsoft Windows software.

Each principal investigator received adjusted questionnaires relevant to all studies in which they were registered as a responsible researcher at the NIHP.

Distribution commenced in July 2022 among 378 principal investigators. Following the death of four principal investigators, their questionnaires were distributed to the second investigator. The questionnaire was distributed in four waves until December 2022. Before the fourth distribution, three additional studies were added to the sample by the NIHP, resulting in a new sample of 381 studies.

### Variables

We collected two types of data: objective metric information of publications presenting finding from the funded studies, along with subjective information reported by the principal investigators in the questionnaires. The measures were analyzed according to the type of organization of each principal investigator that received the funding. The types of organizations included 8 recognized universities in Israel, 15 recognized colleges in Israel, 19 hospitals (comprising 18 general hospitals and one psychiatric hospital), 3 health funds (also known as Health Maintenance Organizations, which insure and provide healthcare in Israel under the national health insurance law), two research institutions, and the Israeli Ministry of Health (MOH).

The objective data included several factors, such as the title of the publication, the journal's name, the publication year, the journal impact factor (JIF) in the past year, the average JIF in the past five years, the journal citation indicator (JCI) in the past year, and the journal’s quarterly index (Q).

JIF was defined as the metric measure for the average citations of the journal's publications over a particular year in one of the two years preceding the year in which the index was published. When the analysis yielded several publications for a certain study with the same JIFs, we chose to analyze the metrics of the earliest publication to reflect the minimum period of time that passed from funding to publication. JCI was defined as the mathematical formula used to calculate the citation impact of a journal's recent publications, weighing citation count and normalizing for different fields of research, which have widely varying rates of publication and citation. The Q ranking estimated in any given category to the quarter that the journal fits in relation to its JIF or JCI. Qs are relative measures of the journal compared to others in a given field of research according to the WOS database. Quarterly data is published in different categories, and for the purpose of the study, we chose the highest Q ranking associated with the journal.

Furthermore, we examined data on the number of citations of the chosen publication for each funded study based on citation metrics presented in two datasets: WOS and Google Scholar. These two databases sometimes yield different citations, as the Google Scholar database often includes citations in journals and scientific sites that are not part of the WOS’s journals.

Objective data was also collected from the questionnaire, including data on publications other than peer-reviewed journals, such as publications in books, chapters in books, lectures in scientific conferences, posters in scientific conferences, independent publications, position papers and opinions, publications in research meetings and seminars, publications in the popular media, and in social media. Investigators were asked to report on policy changes at any level (national, governmental, municipal, institutional, or otherwise) following the study.

The Subjective data collected from the questionnaire offered additional factors to be analyzed, including the target audience of the study (MOH, the Ministry of Finance, the health funds, the hospitals, the clinicians, the scientific community, another), and the impact of the study. The principal investigators were also asked to report on their personal assessment of the extent of the impact of the studies beyond the publications, including the impact on policy making, legislative processes, service organization, and interaction between organizations.

Subsequently, each funded study was categorized by the researchers into seven organizational domains: health policy; health economics; quality of therapy, safety, and equality; technologies; planning, assessment and organization of health services; relationships with factors outside the healthcare system; monitoring and control; and preventive medicine and health promotion. Each study was also categorized into clinical domains. Thirty-four categories were identified, including pharmacy, nephrology, infectious diseases, occupational medicine, orthopedics, gastroenterology, adolescent health, nursing, psychiatry and mental health, gynecology, dentistry, pediatrics, geriatrics, emergency medicine, trauma, genetics, palliative care, cardiology, oncology, neurology, pulmonary medicine, family medicine, otolaryngology, physiotherapy, paramedics, endocrinology, internal medicine, ICU, radiology, transplantation, hematology, gender medicine, ophthalmology, and surgery. Each research study could be assigned to one or more organizational and clinical categories, depending on its title and abstract.

The objective and subjective variables can also be categorized into independent variables, including the research domain, academic degrees of the researchers, their affiliations, year of approval, the institution where the research was conducted, and the amount of funding provided by NIHP; and outcome variables, including the number of peer-reviewed publications, based on both objective and subjective data collected.

### Analysis

Data analysis was conducted during 2023 using descriptive statistics in R System software. Associations between discrete variables were examined using the Chi-Square test, while sequential data were analyzed using ANOVA tests.

All study methods and procedures adhered to acceptable ethical guidelines and regulations. Questionnaires were distributed with an introductory statement, requesting the voluntary cooperation of the investigators. Respondents were given the option to withdraw from participation at any time. The analysis of findings was conducted anonymously.

## Results

Out of the 378 studies initially included in the dataset, 11 were cancelled, and 3 were duplicates. Thus, the final sample consisted of 364 studies funded by NIHP from 2010 to 2020, with a total sum of 55,148,825 NIS. Findings related to these studies were later compared to an internal investigation at NIHP of 459 funded studies from 1996 to 2014, with a total sum of 88 million NIS.

During the study period, approximately 40 studies were approved each year, with an average funding of 151,508 NIS per study (median 138,903 NIS, MIN = 16,500 NIS, MAX = 396,000 NIS, SD = 67,007). All 364 studies were completed within three years. Among these, most studies (313) lasted two years, while 20 were concluded within a year and 31 persisted more than two years.

Table 1 presents the number of funded studies in each period and the average and median funding in NIS, while the years denote the start of each study. The table indicates that the average and median funding remained relatively consistent from 2010 to 2020.

**Table 1:** Number of funded studies and sum of fundings per year

Years	Number of studies	Average (median) budget per research, in NIS
2010–2011	81	163,093 (134,750)
2012–2013	63	146,094 (139,458)
2014–2015	94	151,501 (143,248)
2016–2017	83	160,984 (149,006)
2018–2019*	29	131,235 (121,466)
2020*	14	94,700 (86,353)
Sum	364	151,508 (138,903)

*Incomplete, as some studies were still in progress

Among the principal investigators of the funded studies, 227 held the rank of professor (62%), 113 held a doctoral degree (31%), and the remaining 23 had different academic titles (6%). The majority of the studies were conducted with the involvement of several researchers holding various academic degrees.

The principal investigators were affiliated with 56 institutions and organizations in Israel. Ten percent of these organizations received funding from NIHP for the first time during the years examined. Table 2 displays the distribution of the 364 studies according to the categories of institutions and organizations to which the responsible researchers belong, along with budgetary information. The table reveals that universities and hospitals were the primary institutions leading the list of funded studies. The differences in size of funding according to the type of organization were found to be statistically significant (*p* = .008).

**Table 2:** Funded studies by type of organizations

	Universities	Colleges	Hospitals	Health funds	MOH	Others*	Sum
Number of studies (%)	171 (47)	43 (12)	64 (18)	9 (2)	5 (1)	72 (20)	364 (100)
Average (median) budget, NIS	148,000 (136,300)	139,500 (127,800)	151,600 (138,000)	156,000 (167,300)	94,300 (105,700)	170,293 (162,297)	151,510 (138,900)

*Category of “others” includes 63 studies funded by two research institutions: Gertner (29) and Brookdale (34) (*p* =.008)

The study found a marginally significant correlation between academic rank and the funding of studies. On average, studies led by principal investigators holding the rank of professor received higher funding compared to those led by principal investigators with a doctoral degree without a professorship (156,300 and 142,800 NIS, N = 227, compared to 140,200 and 126,800 NIS, N = 113, *p* = .09).

Categorizing the funded studies according to organizational domains, most studies focused on planning, assessment and organization of health services (*N* = 155, 42.6%) and quality of therapy, safety, and equality (*N* = 134, 36.8%), while studies also focused on health policy (*N* = 76, 20.9%), technologies (*N* = 74, 20.3%), health economics (N = 65, 17.9%), preventive medicine and health promotion (N = 32, 8.8%), relationships with factors outside the healthcare system (*N* = 21, 5.8%), and monitoring and control (*N* = 9, 2.5%). Categorizing the funded studies according to their clinical domains, it was found that the majority of studies focused on psychiatry and mental health (*N* = 36, 10%), geriatrics (*N* = 25, 7%), infectious diseases (*N* = 18, 5%), pediatrics (N = 17, 5%), oncology (*N* = 17, 5%), gynecology (*N* = 15, 4%), and nursing (*N* = 14, 4%).

### Publication characteristics

Out of the 364 funded studies in the sample, 436 publications were retrieved in peer-reviewed journals. Among the funded studies, 137 (38%) resulted in a single publication, 40 (11%) in two publications, 53 (14%) in three or more publications, and 133 (37%) had no peer-reviewed publications.

Table 3 provides a breakdown of the total number of publications per year. The table reveals sporadic changes in the number of publications between years, although these changes were not statistically significant (*p* =.406).

**Table 3:** Number of publications in peer-reviewed journals per year

Years	Number of studies	Total number of publications	Average number of publications per study	Studies with at least one publication	
				N	%
2010–2011	81	127	1.57	62	77
2012–2013	63	81	1.29	43	70
2014–2015	94	91	0.97	60	64
2016–2017	83	99	1.19	47	57
018–2019	29	18	0.62	11	37
2020	14	20	1.43	8	57
Sum	364	436	1.20	231	64

An analysis of publications by type of institution reveals that funded studies conducted in colleges and universities had the highest average number of publications (1.63 and 1.39 publications per study, respectively). This contrasts significantly with studies conducted in health funds, hospitals, and the Ministry of Health (1.11, 0.83, and 1.60 publications per study, respectively), with these differences being statistically significant (*p* = .038). Additional information is provided in Additional File, Table 1.

The average time elapsed from the year of funding to the year of scientific publication was 4.65 years, with a median time of 5 years (MIN = 0, MAX = 10, SD = 2.07). This represents a limited increase compared to the median time of 4.16 years recorded in the previous study for the years 1996–2014.

### Objective measures

We analyzed metric parameters for the top publications of 231 funded studies with at least one publication in peer-reviewed journals. The total percentage of funded studies with at least one publication in the current study for the years 2010–2020 (64%) demonstrates a significant increase compared to data from the previous study conducted in the years 1996–2014 (43%).

The inquiry yielded an average JIF of 5.97 points, and an average number of 7.82 citations according to WOS and an average of 14 citations according to Google Scholar research engine. Analysis reveals that the earliest funded studies in our sample had more publications compared to the latest funded studies (*p* = .0001). Studies with publications received slightly higher funding, with an average of 153,882 NIS, compared to 147,334.9 NIS for studies with no publications; however, these results were not statistically significant (*p* = .371).

Table 4 presents data on JIF, JCI, and the number of citations by type of institution. The table reveals that studies conducted in universities showed higher average impact factors, average JCIs, and citation metrics. However, studies conducted in health funds had higher median impact factors. Nonetheless, these differences were not statistically significant. Additionally, the relationship between JIF and the amount of funding supporting each study was found to be non-significant.

**Table 4:** Publications' metrices by type of institutions

	Universities	Colleges	Hospitals	HMOs	MOH	Others*	Sum	*p*-ANOVA
Number of studies	171	43	64	9	5	72	364	
Number of studies with at least one publication	114	29	40	6	3	39	231	
Percent of studies with at least one publication	67%	67%	63%	67%	60%	54%	64%	
Average (median) JIF (Impact Factor)	6.19 (3.97)	4.21 (3.26)	4.40 (3.84)	4.54 (4.28)	2.76 (2.47)	8.72 (2.69)	5.97 (3.72)	.731
Average JCI (Citation indicator)	1.30	0.98	1.10	1.13	0.78	1.43	1.24	.777
Average number of citations in WOS	9.68	6.34	3.97	3.5	7.67	4.05	7.82	.314
Average number of citations in Google Scholar	17.69	12.07	6.44	13.17	11.67	12.50	14.00	.354

*Category of “others” includes 63 studies funded by two research institutions: Gertner (29 studies, 18 studies with at least one publication) and Brookdale (34 studies, 19 studies with at least one publication).

Examination of the quartile features of journals containing the leading publications of the funded studies indicates that 92 publications appeared in Q1 ranking journals (40%), 64 in Q2 ranking journals (28%), 61 in Q3 ranking journals (26%), and 5 in Q4 ranking journals (2%). Additionally, some studies were published in journals with no Q ranking according to WOS.

Comparing our results to those of the previous study indicates an increase in the average and median JIF, which were lower in the period 1996–2014 (average JIF = 2.73, median JIF = 2.21). However, the number of citations according to WOS was lower, as the studies from 1996–2014 had more time for publishing (average number of citations on WOS = 12.99). JCI cannot be compared since it was not calculated in the previous internal examination.

### Subjective measures

294 of the 367 questionnaires were distributed with an overall response rate (ORR) of 78.2% among the 133 studies that were not published, and 81.9% (*p*= .148) for the 231 published studies. Analyzing the ORR according to the number of publications for each funded study indicate a statistically significant difference (*p* = .002).

Analysis of the questionnaire data allowed for the retrieval of additional publications and dissemination activities, as demonstrated in Additional File, Table 2. The data indicates that the majority of funded studies were presented in peer-reviewed journals and/or lectures.

The questionnaires examined the investigators’ perception of the influences of the funded studies on organizational and regulatory topics. Results are presented in Table 5. The table indicates that nearly half of the principal investigators reported an influence on policy processes (41%), while nearly a third reported influences on the organization of services (28%) and on national/governmental/municipal and/or organizational policies (27%).

**Table 5:** Influences of the funded study reported by investigators (*N* = 293)

Type of influence	N	%
Influence on policy	121	41
Influence on organization of services	83	28
Influence on legislation or support for legislation	20	7
Influence on interrelationships between organizations	54	18
Influence on national/ governmental/ municipal/ and/or organizational policies	78	27

Based on data collected from the questionnaires, a quarter of the principal investigators reported that their studies were published in traditional media and/or social media platforms, such as Facebook and Twitter (25%). Data indicates that funded research in colleges had the highest percentage of media publications (41.7%), followed by funded studies in hospitals (22.4%) and universities (21.9%), while based on the questionnaires, funded studies by the MOH had no media publications, while the differences were statistically significant (*p* = .008). Full data is presented in Additional File, Table 3.

## Discussion

Our study provides an overview of the quality of research funded by the NIHP on health policy in Israel, thereby contributing to the restructuring of the institute’s vision and areas of activity.

A key finding of this study is that 37% of the 364 funded projects did not result in publications in peer-reviewed journals, according to objective data, and 15% were not published on any platform, based on subjective data. We also observed that researchers without prior publications were less likely to complete the questionnaire. Several factors may explain this trend, including lack of time and/or incentives for researchers. The study did not examine the reasons for not publishing, but such data would be valuable for future research.

Future research should also consider response rates and conduct objective analyses, as we did in our study. Furthermore, most studies in this field focus on grants in relation to publications, rather than publications resulting from grants, such as the studies by Boyack & Börner [27] and Khamis et al. [28]. While a few studies examining the number of publications following grants yield varying results, an analysis of grants in spine medical organizations found that 300 grants resulted in 216 peer-reviewed publications (72%), similar to our findings [29]. However, a 20-year study of 4,451 NIH R01 research grants produced a total of 55,053 publications, averaging 12.4 publications per grant [30].

Our current analysis of Israeli-funded research by the NIHP from 2010 to 2020 revealed a twofold increase in the average JIF (5.97) compared to the previous examination spanning 1996 to 2014 (2.73), indicating an enhancement in the objective quality measure of NIHP-funded research. Additionally, the recent study identified a lower number of citations (7.82) compared to the previous examination (12.99). However, these results may be partially attributed to the fact that the previous examination analyzed funded research over a 19-year period, allowing studies from earlier years more time to be published, while the recent study covers only 11 years of funded research. The comparison between the two periods remains valid, considering the almost similar average duration from funding to publication in both the recent (4.65 years) and previous (4.16 years) examinations.

The findings indicate that the research topics funded by NIHP align with the fundamental principles of Israel’s state health insurance law, especially in areas of planning, assessment, and organization of health services, as well as in ensuring the quality of therapy, safety, and equality.

The results are consistent with findings from other studies analyzing the quality of funded research in healthcare. In Lyubarova et al.’s study, an examination of a sample of over 10,000 publications funded by the NIH in the field of cardiac medicine between 1999 and 2006 yielded an average JIF of 5.76, which is similar to the average impact factor obtained in our current study (5.97) [31]. An analysis by De Groote et al. examined 45,716 studies published in 122 scientific journals between 2006 and 2009, of which 7960 were funded by the NIH. The funded studies had an average of 25.2 citations in 2006 and an average of 27.6 citations in 2009, with citation counts measured based on the journal’s report [32]. This figure surpasses the average number of citations received in the current study (an average of 7.82 citations per Web of Science and 14.00 citations per Google Scholar).

To date, extensive work specifically showcasing indices for studies funded in the field of health policy has not yet been published. However, a study from Lebanon examined a sample of 400 publications in 2016 on the topic of health policy and services research, revealing an average JIF of 1.90 [28], which was lower than the one observed in our study. It is important to note that in our current work, we have focused on studies funded by the NIHP, which also involve topics related to health policy in combination with other medical and health domains.

Our study found that a significant proportion of the funded research (77.5%) received mention in the mainstream media, encompassing both traditional and social media platforms, particularly studies conducted in hospitals and universities. These findings potentially indicate a high standard of funded research, aligning with the observations by Anderson et al., who noted that scientific studies receiving greater exposure in non-scientific media outlets, such as mainstream news and social media, tend to garner more citations in peer-reviewed scientific literature [33]. However, a study conducted by Selvaraj et al. found no direct correlation between media coverage and research quality, highlighting newspapers’ inclination to prioritize observational studies over randomized controlled trials and to select studies of varying quality [34].

Several studies have investigated the impact of research grants on publication metrics. Lyubarova et al. found that research funded by the NIH was accompanied by publications with a higher JIF compared to non-funded studies by NIH [31]. Another analysis of U.S. studies identified a positive correlation between funding sources and an increase in the number of citations of articles [35]. A study in Japan revealed that funded studies result in publications with more citations, a higher citation index, and a higher JIF [36]. An examination from Hungary of research proposals submitted to the National Institute for Research in the country during 2006–2015 found that funded studies were accompanied by more publications in top-ranked journals (Q1) [37]. However, our study sample consisted solely of research funded by the NIHP, preventing us from examining such connections.

The study has several limitations. First, the dataset included only studies funded by the NIHP, so our analysis could not be broadened to include non-funded studies. Conducting such an examination in the future could provide additional insights into the topic of quality assessment of funded research. Second, the questionnaire used in our study may introduce a selection bias, as investigators in studies with publications may have had a higher incentive to respond. However, the response rate was quite high (ORR = 79.8%), and the findings were also supported by our research conducted across three datasets to search for publications. Additionally, the overall rate of funded studies with no publication in peer-reviewed journals was similar between the objective analysis (34%) and the subjective reporting in the questionnaires (38%), suggesting that both methods yielded comparable findings. Third, it is important to note that this study was initiated by the NIHP, which decides and distributes funding, and this may have influenced the responses in the questionnaires from investigators. However, our analysis was done anonymously in order to reduce such effects. Fourth, the comparison of our results to a previous internal examination of studies funded by NIHP from 1996 to 2014 introduces a four-year overlap with the current analysis of studies from 2010 to 2014. While this overlap may be considered a limitation, the comparison remains valuable for examining trends in the influence of NIHP-funded studies over more than two decades. Fifth, changes in publication metrics should be considered with caution due to the evolution of publications over time, particularly with the digitalization of articles, which offers greater opportunities for dissemination and may lead to increased citations, thereby influencing journals' impact factors. Sixth, the distribution of studies across disciplines may affect JIF and citation data. Seventh, since metrics were collected at a specific point in 2022, earlier-published studies would have had more time to accumulate citations. Eighth, our retrospective analysis could not assess the actual impact of NIHP-funded studies on policy. However, we present the researchers’ subjective perceptions of policy impact, as shown in Table 5 and Additional File, Table 3.

Based on the findings of our study, several recommendations emerge to enhance the quality and impact of future funded studies. Our analysis platform uses the quality of publications as a factor influencing the potential impact of study quality and as a surrogate for assessing the overall quality of the study. Therefore, we suggest that health policy funding organizations implement a follow-up reporting system, to be submitted 1–2 years after study completion, to gather data on publications in scientific journals and other relevant platforms. Some funders require the reporting of publications, such as NIHR and research councils in the UK. Furthermore, we advocate for funding agencies to review the prior publications of principal investigators in research proposals and to explore alternative methods for promoting the dissemination of funded studies. The NIHP took such a step in 2018 by allocating an additional budget to cover publication fees for funded studies.

Following the available scientific literature on the topic, we also recommend that the National Institute consider, initiate, and foster collaborations among researchers from diverse fields and organizations for specific research proposals submitted to the Institute. Engaging experts from various disciplines with a multidisciplinary approach may demand additional time and effort, but it facilitates diverse analysis and could ultimately enhance the quality of research in the field of health policy [38].

## Conclusions

The findings of the study illuminate the scope and quality of research activities funded by the NIHP over a decade, from 2010 to 2020. In both aspects—scope of activity and fundamental publication metrics—the findings indicate an increase compared to the previous period examined (1996–2014).

The study emphasizes the importance of various research outputs beyond publication metrics in scientific journals. These outputs, including other types of publications, the impact of research on policy management aspects, their portrayal in the media, and their role in training a new generation of researchers, may be invaluable to the healthcare system.

While assessing the impact of research products on decision-makers presents challenges, the analysis introduced in our study offers a method for evaluating the quality of publicly funded studies in health policy. This approach may facilitate the prioritization of funding for such studies in the future.

## Supplementary Information

Additional file1.

## Data Availability

The dataset used and/or analyzed in the current study is available from the corresponding author on reasonable request.

## Abbreviations

JCI: Journal citation indicator
JIF: Journal impact factor
MOH: Israeli ministry of health
NIHP: The Israeli National Institute for Health Policy Research
NIS: New Israeli shekels
ORR: Overall response rate
Q: Quarterly index
U.K: United Kingdom
U.S.: United States of America
WOS: Web of science
