# Supplementary material for: Assessing the quality of studies funded by the Israel National Institute for Health Policy Research, 2010–2020
Source: Isr J Health Policy Res. 2025 Mar 5;14:10. doi: 10.1186/s13584-025-00672-w (PMC11881469; doi:10.1186/s13584-025-00672-w)
Supplement: Supplementary file 1 — Additional file1. [file 13584_2025_672_MOESM1_ESM.docx]

**Additional File**

**Title:**

**Assessing the Quality of Studies Funded by the Israel National Institute for Health Policy Research, 2010-2020**

Dan Even, Moshe Leshno & Avi Porath

**Table 1: Publications by Type of Institution**

ראש הטופס

|  | **Universities** | **Colleges** | **Hospitals** | **Health funds** | **MOH** | **Others*** | **Sum** |
| --- | --- | --- | --- | --- | --- | --- | --- |
| **Number of studies** | 171 | 43 | 64 | 9 | 5 | 72 | 364 |
| **Total number of publications** | 238 | 70 | 53 | 10 | 3 | 62 | 436 |
| **Average number of publications per study** | 1.39 | 1.63 | 0.83 | 1.11 | 0.6 | 0.86 | 1.20 |

* Category of "others" includes 63 studies funded by two research institutions: Gertner (29) and Brookdale (34) (p=.038)

**Table 2: Types of Publications and Dissemination Activities Reported by Investigator (N=293)**

ראש הטופס

| **Type of publication/ Dissemination activity** | **N** | **%** |
| --- | --- | --- |
| Any type of publication | 250 | 85 |
| Publication in peer-reviewed journals | 183 | 62 |
| A chapter in a book | 21 | 7 |
| A lecture in a conference | 184 | 63 |
| A poster in a conference | 71 | 24 |
| An independent publication, a policy statement and/or an opinion | 38 | 13 |
| Graduate thesis (MA, PhD, etc.) | 126 | 43 |
| Presented in meetings and seminars | 228 | 78 |

**Table 3: Publications in the traditional and social media by type of institution, reported by Investigators (N=293)**

|  | **Universities** | **Colleges** | **Hospitals** | **Health funds** | **MOH** | **Others*** | **Sum** |
| --- | --- | --- | --- | --- | --- | --- | --- |
| **Number of studies** | 171 | 43 | 64 | 9 | 5 | 72 | 364 |
| **number of Questionnaires** | 151 | 36 | 49 | 5 | 4 | 48 | 293 |
| **Number of studies published in the traditional and/or social media** | 33 | 15 | 11 | 1 | 0 | 14 | 74 |
| **Percentage of Studies published in the traditional and/or social media** | 21.9 | 41.7 | 22.4 | 20.0 | 0.0 | 29.2 | 25.3 |

Category of "others" includes studies funded by two research institutions: Gertner (19 questionnaires were filled) and Brookdale (23 questionnaires were filled) (p=.008)
